# Cell cycle regulation in *Trypanosoma brucei*

**DOI:** 10.1016/j.molbiopara.2007.01.017

**Published:** 2007-05

**Authors:** Tansy C. Hammarton

**Affiliations:** Division of Infection & Immunity and Wellcome Centre for Molecular Parasitology, University of Glasgow, Biomedical Research Centre, 120 University Place, Glasgow G12 8TA, United Kingdom

**Keywords:** BSF, bloodstream form, PCF, procyclic form, CDK, cyclin-dependent kinase, CRK, cdc2-related kinase, MAPK, mitogen-activated protein kinase, RNAi, RNA interference, BB, basal body, IFT, intraflagellar transport, N, nucleus, K, kinetoplast, kDNA, kinetoplast deoxyribose nucleic acid, VSG, variant surface glycoprotein, FAZ, flagellum attachment zone, APC, anaphase promoting complex, MEN, mitotic exit network, FEAR, Cdc14 early anaphase release network, Rb, retinoblastoma, *Trypanosoma brucei*, Trypanosomatid, Cell cycle, Signal transduction, Mitosis, Cytokinesis

## Abstract

Cell division is regulated by intricate and interconnected signal transduction pathways that precisely coordinate, in time and space, the complex series of events involved in replicating and segregating the component parts of the cell. In *Trypanosoma brucei*, considerable progress has been made over recent years in identifying molecular regulators of the cell cycle and elucidating their functions, although many regulators undoubtedly remain to be identified, and there is still a long way to go with respect to determining signal transduction pathways. However, it is clear that cell cycle regulation in *T. brucei* is unusual in many respects. Analyses of trypanosome orthologues of conserved eukaryotic cell cycle regulators have demonstrated divergence of their function in the parasite, and a number of other key regulators are missing from *T. brucei*. Cell cycle regulation differs in different parasite life cycle stages, and *T. brucei* appears to use different checkpoint control strategies compared to model eukaryotes. It is therefore probable that *T. brucei* has evolved novel pathways to control its cell cycle.

## 1 Introduction

**Fig. 1:**
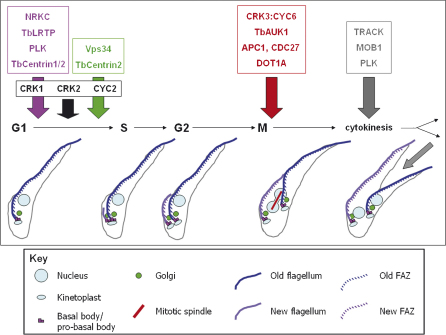
Cell cycle regulation in *Trypanosoma brucei*. Duplication of the major organelles and structures during the cell cycle is illustrated in cartoon format for the PCF. Experimentally verified regulators of G_1_ phase are shown in black, basal body duplication in pink, Golgi duplication in green, mitosis in red and cytokinesis in grey. Adapted from [2].

The typical eukaryotic cell cycle consists of four phases—G_0_/G_1_, S, G_2_ and M. During the first gap phase (G_0_/G_1_), the cell prepares for entry into a new round of replication and cell division, and ensures the availability of nutrients and frequently, also the cell size, are appropriate to proceed to S phase. DNA is replicated during S phase, and following the second gap phase (G_2_), is divided during M phase. In animal cells, cytokinesis commences before mitotic chromosome segregation is completed, and hence the two events overlap. Although the cell division cycle in *Trypanosoma brucei* broadly follows this scheme, it possesses unique features and complexities [1,2]. The parasite is vermiform in shape, a property conferred by the sub-pellicular microtubule corset of the cytoskeleton. *T. brucei* contains a number of single copy organelles and structures (e.g. nucleus, mitochondrion whose DNA is concentrated into a disc-like structure termed the kinetoplast, Golgi and basal body/flagellum complex), which must be accurately duplicated and segregated if cell division is to generate viable progeny. Organelle duplication therefore occurs in a precise order (Fig. 1, [1]). The duplicated organelles are concentrated in the posterior end of the cell (although their relative positioning differs in different parasite life cycle stages), imposing constraints on cytokinesis, which occurs after mitotic chromosome segregation via the unidirectional ingression of a cleavage furrow along the helical axis of the cell from the anterior to the posterior end.

## 2 Cell cycle regulators

Molecular regulation of the *T. brucei* cell cycle has unique and unusual features, reflecting the complexities seen at the physical level. The publication of the so-called ‘TriTryp’ (*T. brucei*, *Trypanosoma cruzi* and *Leishmania major*) genome sequences [3–5] has greatly impacted trypanosomatid cell cycle research, leading to faster functional analyses particularly in *T. brucei* where RNA interference (RNAi) is possible, and allowing the description of the trypanosomatid kinomes [6]. Orthologues of many conserved protein kinases, such as cyclin-dependent kinases (CDKs), mitogen-activated protein kinases (MAPKs), aurora and polo-like kinases, are present in *T. brucei*, although their functions are often divergent [7–9], and may also differ in different life cycle stages. Conversely, no receptor-linked tyrosine kinases were found; tyrosine phosphorylation is likely carried out by dual specificity protein kinases. Additionally, some cell cycle checkpoints are known to be absent in particular life cycle stages of *T. brucei* [1,7], and some checkpoint regulators such as the spindle checkpoint protein BUB1, centromeric histone (CenH3), and Rho GTPases, are also apparently absent in the trypanosomatids.

### 2.1 CDKs

CDK activity is essential for progression through different cell cycle boundaries, with different CDKs interacting with different cyclins to regulate different cell cycle stages [10]. For example, in mammalian cells, CDK4 and CDK6 are activated by interaction with cyclin D (transcribed in response to mitogenic signals) and regulate G_1_ progression by inhibiting the retinoblastoma (Rb) protein, which itself represses transcription of the S phase cyclin, cyclin E. Cyclins E and A complexed with CDK2 are required for S phase, promoting centrosome duplication and DNA replication, while mitosis is regulated by CDK1 complexed with cyclins A and B. There is clear evidence that trypanosomatid cell cycles are also regulated by CDKs [1,2,11], although modulation of CDK activity may have evolved trypanosomatid-specific features. *T. brucei* possesses eleven cdc2-related kinases (CRK1-4 and CRK6-12) [6] (Table 1). CDKs are activated by the binding of a cyclin partner, and *T. brucei* contains 10 cyclins, CYC2-11 (Table 2). Alternative names have been proposed for some of the *T. brucei* cyclins (Table 1) [12], but these are somewhat misleading, as, with the exception of CYC6/CycB2, functional equivalence to mammalian B or E-type cyclins has not been demonstrated. Unfortunately, little is known about the pairings of these cyclins and CRKs, and to date, only CRK3 in *T. brucei* has been demonstrated to be cyclin-dependent, interacting with both CYC2 and CYC6 [7,11].

In addition to being regulated by cyclin binding, the activity of CDKs is modulated by phosphorylation of conserved residues. Phosphorylation of the T-loop threonine residue, T161 (numbering for human CDK1) by the CDK-activating kinase (CAK) increases the activity of the CDK:cyclin complex, while phosphorylation of T14 and Y15 by Wee1 and Myt1, respectively, inhibits the activity. In mammals, CAK comprises CDK7, MAT1 and cyclin H subunits, but although trypanosome CRK7 displays some similarity to CDK7, MAT1 and cyclin H homologues have not been identified. Homologues of Wee1 kinase are present in the trypanosomatids [6], and trypanosomatid CRKs associate with the CDK accessory protein homologue, CKS1 [7,11], but homologues of Myt1, Cdc25 (the phosphatase that removes the inhibitory T14 and Y15 phosphorylations), Tome1, (an SCF type-E3 ligase that targets Wee1 for degradation at the onset of mitosis) and CDK inhibitor proteins (CKIs) have not been found. The lack of these regulators is potentially explained by the divergence observed in CRK primary amino acid sequence (Table 1). Although all CRKs cluster phylogenetically with CDKs from other organisms [6], over half contain insertions in their kinase catalytic domain compared to human CDK1, and most have extensions at the N- and/or C-terminus. The PSTAIRE box motifs (site of cyclin binding), although recognisable, are divergent, and a number of CRKs have amino acid substitutions at T161, T14 and/or Y15. This may suggest that other mechanisms have evolved to regulate the activity of CRKs in trypanosomatids.

### 2.2 Phosphatases

Protein phosphatases also play crucial cell cycle regulatory roles. For example, in mammals, PP2A regulates sister chromatid cohesion and mitotic exit, PP1 regulates chromosome architecture and chromatin structure during mitosis, CDC25 phosphatases activate CDKs, and CDC14 phosphatases are implicated in centrosome maturation, spindle stability, mitotic exit and cytokinesis (reviewed in [13]). In *T. brucei*, approximately 30 putative protein phosphatases have been annotated at GeneDB (www.genedb.org), suggesting dephosphorylation is also likely to play important roles in cell cycle regulation in this organism. In support of this, okadaic acid (an inhibitor of protein phosphatases PP1, PP2A and PP2B) inhibits kinetoplast segregation in procyclic *T. brucei* [14], and apparently can reverse the cell cycle arrests that occur upon downregulation of certain CRKs by RNAi [15].

## 3 Regulation of G_1_ phase

During G_1_ phase, cells sense environmental conditions to determine whether to proliferate, differentiate, quiesce or undergo apoptosis. In mammalian cells, the ERK/Ras and PI3K pathways act cooperatively to promote G_1_ progression through CDK activation in response to signals transduced through receptor-linked tyrosine kinases or G-protein coupled receptors following growth factor stimulation [16]. Available evidence suggests that trypanosomes regulate G_1_ phase progression through different mechanisms. Receptor-linked tyrosine kinases and G-protein coupled receptors are absent from these organisms, MAPKs have not been implicated in G_1_ progression to date, and *T. brucei* seems to possess just one genuine PI3K, Vps34, which is required for Golgi segregation, but apparently not for progression through G_1_ [17].

*T. brucei* CRKs are, however, required for progression through G_1_ phase. RNAi of *CYC2* arrested both bloodstream (BSF) and procyclic form (PCF) *T. brucei* in G_1_ phase [12,18], and CYC4/CycE3 may also be required for G_1_ progression [12]. Although CYC2 is known to activate CRK3, it cannot be ruled out that its G_1_ role arises from its interaction with another CRK, as a full analysis of CRK:cyclin pairings has not yet been completed. Depletion of CYC2 also resulted in a morphogenic phenotype in the PCF, characterised by active microtubule extension at posterior end of the cell, generating an elongated ‘nozzled’ morphology [18]. This morphological defect had previously been observed in G_1_ cells following overexpression of a zinc finger protein, ZFP2, either during the stumpy to procyclic differentiation process, or in the PCF itself [19], arguing that control of morphogenesis and G_1_ phase progression are tightly linked in this life cycle stage. RNAi of *CRK1* in both BSF and PCF life cycle stages also enriched cells in G_1_ [20], and this phenotype was enhanced by co-downregulation of *CRK2* [21]. Simultaneous depletion of CRK1 and CRK2 (but not depletion of either protein individually) also caused a PCF-specific morphological defect. Cells with extended posterior ends were observed, and in some cases, bifurcation of the elongated posterior occurred. It will be interesting to discover the cyclin partners of CRK1 and CRK2, as previous work indicated that CYC2 did not interact with either of these CDKs [22].

### 3.1 Regulation of other G_1_ events

#### 3.1.1 Basal body and flagellum duplication

Duplication of the basal body (BB) and outgrowth of the daughter flagellum are the earliest detectable cytological events during the trypanosome cell cycle, occurring at 0.41 of the unit cell cycle in PCF *T. brucei* (approximately 3.5 h after the start of G_1_ phase, given an average cell cycle of 8.5 h) [23]. As well as γ- and δ-tubulin [24], a number of proteins have been localised to the BB: TbCentrin1 and TbCentrin2, required for BB duplication [25]; TbLRTP, a negative regulator of BB duplication and flagellum biogenesis [26], and NRKC, a NIMA-related kinase, that may activate separation of the mother and newly-matured basal bodies [27]. More recently, polo-like kinase (PLK) has also been shown to be required for BB duplication, since its depletion by RNAi in PCF *T. brucei* generated aberrant cells containing two nuclei but only one kinetoplast, one BB and one flagellum [56]. However PLK does not appear to localise to the basal bodies, and may therefore act as an upstream regulator of BB duplication. The flagellum is built by means of both intraflagellar transport (IFT) (see [28] for a comprehensive review of the trypanosome flagellum) and non-IFT-dependent transport [29]. MAPKs regulate flagellum length in *Leishmania* [30] and the PACRG proteins are required to maintain outer microtubule doublets along the trypanosome axoneme [31].

#### 3.1.2 Golgi duplication

Duplication of the Golgi apparatus in PCF *T. brucei* commences just after BB duplication and takes about 2 h to complete. The new Golgi appears *de novo*, close to the new endoplasmic reticulum export site, and is at least partly constructed from materials derived from the old Golgi [32]. In addition to localising to the BBs, TbCentrin2 associates with the Golgi as a bi-lobed structure, and is required for its duplication, possibly defining the site of new Golgi construction [25]. Golgi assembly is an ordered process, and initial data indicate that structural and enzymatic components are assembled prior to other components required for transporting and sorting cargo [33]. However, the signals for initiation of Golgi duplication are currently unknown. The Golgi is segregated alongside the basal bodies and kinetoplast, a process that requires Vps34 [17]. Depletion of Vps34 in BSF *T. brucei*, in addition to impairing membranous endocytic trafficking, inhibited Golgi segregation without affecting basal body separation, suggesting this kinase is required for the coupling of Golgi and basal body segregation.

## 4 S phase

The *T. brucei* nuclear genome comprises 11 megabase chromosomes, several intermediate chromosomes (∼200–900 kb) and approximately 100 minichromosomes (∼50–150 kb), while the mitochondrial genome contains several thousand minicircles (∼1 kb) and a few dozen maxicircles (∼23 kb). Replication of the genomes commences approximately 3 h into the cell cycle (in PCF *T. brucei*) and takes about 2 h to complete. Kinetoplast S phase starts just before nuclear S phase, and this defined period of mitochondrial DNA replication contrasts with mammalian cells where the DNA of multiple mitochondria is replicated throughout the cell cycle. The complex process of minicircle replication has been studied in detail and recently reviewed [34]. The kDNA is surrounded by its own precisely positioned replication machinery, and so far around 30 components of this machinery are known, but it is likely many more remain to be identified [35]. However, the mechanisms of maxicircle replication and the signalling events that initiate and license kDNA S phase are poorly understood.

Nuclear DNA replication is also, somewhat surprisingly, a poorly studied process in trypanosomes. In mammals, a pre-replication complex consisting of the hexameric origin recognition complex (ORC, comprising ORC1–ORC6 subunits), CDC6 and Cdt1 forms at origins of replication during G_1_ phase (reviewed in [36]). During the transition to S phase, these licensing factors recruit the MCM2-7 complex, which possesses helicase activity to promote the unwinding of DNA at the origin. MCM10 is recruited, which in turn recruits CDC45, another factor essential for unwinding of the origin and loading of polymerases. Conversion of the pre-replication complexes into replication forks requires the activity of S phase CDK and DDK, a Dbf-dependent kinase consisting of the CDC7 kinase and its activating partner DBF4. MCM10 facilitates DDK to phosphorylate MCM2-7, which activates the complex's helicase activity. Finally, DNA polymerases are loaded. The TriTryp genome sequences [3] indicate that the replication fork synthesis machinery from higher eukaryotes is conserved, but only one subunit of the origin recognition complex, ORC1/CDC6, is present; other replication initiation proteins, including MCM10, Cdt1 and DBF4, appear to be absent also, suggesting that initiation of DNA replication in trypanosomatids may resemble that of the Archaea. In other eukaryotes, re-replication of DNA within a single cell cycle is prevented by rising CDK levels, which ensure the licensing machinery is downregulated from S phase until the M-G_1_ transition, and also, in higher eukaryotes by the proteolysis and geminin-mediated inhibition of Cdt1 following S phase initiation [36]. Following the downregulation of mitotic CDK at the end of mitosis, licensing of origins begins again in preparation for DNA replication in the next cell cycle. Published functional data for the DNA replication machinery in *T. brucei* does not yet exist, but inhibiting cytokinesis by a variety of mechanisms results in repeated DNA re-replication [1,2], suggesting that timing of cell division plays an important role in ensuring that DNA is not re-replicated in a single cell cycle. Additionally, DNA re-replication occurs asynchronously in the two nuclei, since cells with odd number of nuclei (e.g. 3N, 5N) are generated [37], suggesting that the nuclei are re-licensed for replication at different rates.

## 5 G_2_ and M phase

Progression through the G_2_/M phase transition in eukaryotes requires CDK1-cyclin B activity, which increases during G_2_ phase as a result of Tome-1 mediated targeting of Wee1 for degradation and CDC25 phosphatase activity. Activation of CDK1 leads to activation of the anaphase promoting complex (APC), which ubiquitinates anaphase inhibitors, targeting them for degradation by the 26S proteosome, thereby triggering progression from metaphase to anaphase. Entry into anaphase is delayed by the spindle assembly checkpoint, which inhibits the APC, until spindle assembly is complete and all chromosomes are correctly attached [38]. Additionally, the cohesin complex, which acts as a molecular glue to hold sister chromatids together from S phase to anaphase, must be cleaved by the protease separase, to enable sister chromatid segregation. Exit from mitosis is controlled by the mitotic exit network (MEN) and Cdc14 early anaphase release (FEAR) cascades [38].

The trypanosome G_2_/M phase transition is also regulated by the activity of mitotic CDK. *T. brucei* CRK3 (the functional homologue of mammalian CDK1) complexed with CYC6 is required for mitosis [7,12,20]. Downregulation of either of these proteins by RNAi prevented mitosis, although for CRK3, its downregulation might have been expected to have arrested cells in G_1_ as well as M phase since it interacts with CYC2, but this was not observed [20]. Differential phenotypes are seen in BSF and PCF cells, because PCF, but not BSF, cells lack a mitosis to cytokinesis checkpoint. Therefore, in BSF *T. brucei*, inhibition of mitosis prevents cytokinesis, although not re-replication of nuclear and kinetoplast DNA, or segregation of re-replicated kinetoplasts, resulting in cells with a single enlarged nucleus and multiple kinetoplasts. In the PCF, however, cytokinesis occurs despite the inhibition of mitosis, resulting in a cell with 1 nucleus (with replicated DNA) and 2 kinetoplasts (1N*2K) dividing to generate asymmetric daughter cells; one with a tetraploid nucleus and a kinetoplast (1N*1K), and one cell containing just a kinetoplast (termed a zoid, 0N1K). Recently, RNAi knockdown of the aurora-like kinase, TbAUK1, was shown to inhibit mitotic spindle assembly in PCF and BSF *T. brucei*, and was reported to inhibit cytokinesis in PCF as well as BSF cells [39,40], suggesting that TbAUK1 may also be required to initiate cytokinesis, at least in PCF cells. Ectopically expressed HA-tagged TbAUK1 localised to the nucleus in 1N1K cells, but relocated to the spindle at mitosis and to the spindle midzone during late mitosis, consistent with a spindle assembly function. Mammalian aurora B kinase is a component of the spindle assembly checkpoint, but it is not yet known whether such a checkpoint operates in *T. brucei*.

The similarity in mitotic phenotypes observed upon RNAi of *CYC6*, *CRK3* and *AUK1* suggests that AUK1 and CRK3:CYC6 may function in the same mitotic regulatory pathway. However, the distinctive phenotypes seen in different life cycle stages raise some interesting questions about the regulation of mitosis. In PCF *T. brucei*, following RNAi of *AUK1*, *CYC6* or *CRK3*, the nucleus in 1N2K cells became elongated, but never bilobed, and in the case of *AUK1* RNAi, it was demonstrated that the nucleoli did not segregate [40]. However, in BSF 1N>2K cells, nuclei were frequently observed to become bi-lobed or multi-lobed, and segregation of the nucleoli was demonstrated following *AUK1* RNAi [40]. It is therefore possible that these regulators act later in mitosis in BSF cells compared to the PCF. A similar differential effect was reported following downregulation of putative anaphase-promoting complex (APC) components APC1 and CDC27 [41]. In the PCF, 1N*2K cells with short metaphase-like mitotic spindles accumulated before dividing to generate 1N*1K and 0N1K daughters, while in the BSF, 2N2K cells accrued in which the two nuclei were still connected by an elongated anaphase-like spindle structure. This may suggest that APC components function earlier in mitosis in PCF cells than in the BSF. However, it is difficult to separate the contributions of molecular regulation and timing of cytokinesis, given the lack of a mitosis to cytokinesis checkpoint in PCF *T. brucei*; it is possible that PCF cells would also progress to anaphase (perhaps because of residual APC activity following RNAi) if cytokinesis was inhibited. It can also not be ruled out at present that differences in residual APC activity following RNAi account for the stage-specific differences. However, it seems probable that *T. brucei* has evolved novel molecular mechanisms to regulate mitosis (probably due to the distinctive structural features of mitosis, see below), and it is notable that homologues of MOB1, part of the MEN in yeast, and PLK, which plays multiple mitotic roles in many organisms, do not appear to regulate parasite mitosis [8,9].

The physical mechanisms of mitosis in *T. brucei* are distinctive and complex, given that the organism must segregate around 240 chromosomes following DNA replication. Putative kinetochores (about eight per cell) have been visualised by electron microscopy, along with pole-to-pole and kinetochore microtubules, although there are clearly not enough kinetochores to segregate even the megabase chromosomes by a single chromosome: kinetochore microtubule mechanism [42]. Minichromosomes segregate with different kinetics compared to megabase chromosomes, and it is proposed that minichromosomes segregate laterally along pole-to-pole microtubules before megabase chromosomes are segregated via a kinetochore-dependent mechanism, possibly involving the attachment of more than one chromosome to each kinetochore [42]. The trypanosomatid kinetochore is likely to be unique in terms of its protein constituents, as no homologues of the centromeric histone, CenH3, or of the centromeric checkpoint protein, BUB1, have been identified [3]. Structural differences in chromatin, perhaps because of differences in histone H1 modifications, have also been noted in BSF and PCF *T. brucei* [43] and defects in histone modification lead to problems in mitosis. For example, deletion of the histone deacetylase gene, *DAC4*, in BSF *T. brucei* enriches the proportion of 1N2K cells within the population, suggesting a delay in mitotic progression [44]. More recently, dimethylation of histone H3 at K76 has been shown to be a marker for mitosis, and downregulation of the dimethylase responsible for this modification, DOT1A, in BSF *T. brucei* generated cells with haploid DNA content, suggesting cells had undergone an additional round of mitosis and cytokinesis without first replicating their DNA [45], resembling (superficially, at least) the cell division of meiosis II. Apparent orthologues of some components of the cohesin complex (SMC1, SMC3 and SCC1/RAD21 but not SCC3), as well as separase, which cleaves the SCC1 subunit at anaphase to enable sister chromatid segregation, are encoded by the *T. brucei* genome. Additionally, the *T. brucei* genome encodes putative orthologues of the condensin subunits SMC2, SMC4 and CAP-D2, which are required for the condensation of chromatin in other organisms. However, given that nuclear DNA does not condense at mitosis in *T. brucei* [46], the significance of this is unclear.

## 6 Cytokinesis

Cytokinesis can be considered to consist of four main events: cleavage site selection, a signalling event(s) to initiate cleavage, furrow ingression and cell abscission. Cytokinesis in *T. brucei* is mechanistically very different from the classical actomyosin ring constriction seen in animal cells, and occurs after nuclear DNA segregation is complete, but can still be considered in terms of these events.

### 6.1 Cleavage site selection

The site of furrow initiation in *T. brucei* is determined by the anterior end(s) of the newly-synthesised flagellum and/or flagellum attachment zone (FAZ), as intraflagellar transport mutants, which produce short new flagella undergo cytokinesis with the furrow initiating too close to the posterior end of the cell [47]. However, the molecular determinants of cleavage site selection are currently unknown.

### 6.2 Signalling cytokinesis

Little is currently known about what triggers entry into cytokinesis in *T. brucei*. Many mutant cell lines accumulate enlarged cells containing multiple nuclei and kinetoplasts, indicating that cytokinesis is blocked, allowing re-replication of DNA to occur, but in the majority of cases, it is likely the cytokinesis defect is an indirect effect. For example, downregulation of proteins required for flagellum attachment, basal body duplication and protein GPI anchor biogenesis inhibits cytokinesis, but it is unlikely that any of these proteins play direct roles in initiating cytokinesis [1,2,27]. Similarly, it has been reported that downregulation of PLK in PCF *T. brucei* prevents initiation of cytokinesis [8], but more recent data indicates that this most likely arises because of a prior inhibition of basal body duplication (see above). APC components have been shown to be required for the latter stages of mitosis [41], and it is possible that they may provide a link between mitosis and cytokinesis. TbAUK1 and TRACK (trypanosome receptor for activated C kinase) may be involved in initiating cytokinesis in the PCF and BSF, respectively [37,39], although TRACK plays a more specific role in furrow ingression in procyclic cells (see below). Furthermore, RNAi of variant surface glycoprotein (VSG) in BSF trypanosomes resulted in a rapid pre-cytokinesis block; 2N2K cells accumulated, but no re-replication of DNA was observed, suggesting a specific checkpoint is invoked upon depletion of VSG transcripts or protein [48], which must normally be inactivated for entry into cytokinesis in this life cycle stage. It has also been proposed that mitochondrial fission inactivates a cytokinesis checkpoint, since RNAi ablation of dynamin-like protein (DLP) in PCF cells, as well as inhibiting mitochondrial fission and endocytosis, blocked the latter stages of cytokinesis, without leading to re-replication of DNA [49].

### 6.3 Furrow ingression

Furrow ingression in animal cells involves the constriction of an actomyosin ring as well as vesicle trafficking to the furrow to provide additional membrane. However, there is currently no evidence to support the involvement of an actomyosin ring in *T. brucei* cytokinesis. Indeed, furrow ingression is unidirectional and helical, and it is possible that the microtubule-based cytoskeleton may necessitate specialist mechanisms to effect cytokinesis. To date, the only identified role for actin in *T. brucei* is in endocytosis in BSF trypanosomes; in PCF cells, a 10–20-fold downregulation of actin protein by RNAi led to distortions in the *trans*-Golgi network without affecting growth [50]. Functional studies of *T. brucei* myosins have not been carried out to date, and the parasite also appears to lack septins, which localise to the cleavage site in yeast, as well as homologues of Rho GTPases (e.g. RhoA, Cdc42), their activators (e.g CYK4, IQGAP) and substrates (e.g. Rho kinase), which play crucial roles in regulating cytokinesis in other eukaryotes. Vesicle trafficking to the furrow has not been reported to date, and it is possible that *T. brucei* synthesises sufficient membrane to form two daughter cells prior to cytokinesis initiation, and just remodels it during furrowing.

Furrow ingression in *T. brucei* may therefore be controlled by novel regulators and effectors, and evidence is accumulating that BSF and PCF life cycle stages regulate this process differently. In the BSF, downregulation of MOB1 results in an accumulation of post-mitotic cells with a visible cleavage furrow [9], indicating that this protein is required for furrow progression, and suggesting that furrow ingression in BSF *T. brucei* comprises MOB1-independent and dependent phases. MOB1 interacts with the NDR family protein kinase, PK50, which is a functional homologue of the Orb6 protein kinase that regulates cell morphology and cell cycle progression in yeast [9,51]. In the PCF, downregulation of TRACK also prevents completion of furrow ingression, and over time, partially furrowed cells go on to re-duplicate their organelles and re-initiate cytokinesis, resulting in aggregates of partially divided cells [37]. In contrast to the BSF, MOB1 appears to be required for accurate furrowing, rather than for furrow ingression per se in procyclic cells [9]. Notably, none of these regulators have been localised to the cleavage furrow, but have been reported to be cytoplasmic [9,51], although TRACK is additionally enriched at the nuclear periphery [37], and the localisation of PLK is controversial having been localised to the cytoplasm in one study [56] and the FAZ in another [8]. This may suggest that these proteins regulate, as yet unidentified, downstream effectors, which do localise to the furrow.

### 6.4 Abscission

Following furrow ingression, *T. brucei* cells remain joined at their posterior ends for some time before separating, but to date, no proteins have been localised to this site or demonstrated to be functionally involved in the final step of cytokinesis. However, flagellar beat contributes to abscission in PCF *T. brucei* [52], and is essential for initiation of cytokinesis in bloodstream cells [53,54].

## 7 Concluding remarks

Significant advances in our understanding of cell cycle control in *T. brucei* have been made over the past few years, helped considerably by the widespread application of RNAi in phenotype analysis and the publication of the genome sequence. It is now well established that there are significant differences in cell cycle regulation between the parasite and its mammalian hosts, and hopefully, some of these differences will be exploitable in the future in order to develop new anti-parasitic drugs. The cell biology of the trypanosome is highly intriguing, with the discoveries that cell cycle regulation differs in different life cycle stages of the parasite, mitosis and cytokinesis appear to be regulated separately, many important eukaryotic cell cycle regulators are not conserved and that the parasites have evolved unique checkpoint strategies.

However, there are considerable challenges ahead, made all the more difficult by our continued inability to reliably synchronise *T. brucei* in any given cell cycle phase. One of the key challenges will be to link individual regulators in pathways and discern their order in a pathway. There is also a need to determine which regulators control the transition from one cell cycle phase to the next; for example, does a given mitotic regulator control entry into mitosis, events during mitosis, or exit from mitosis and entry into cytokinesis? More detailed and precise functional studies, as well as information about the substrates and activators of a given regulator will be needed to resolve these questions. Many cell cycle regulators undoubtedly remain to be identified. Forward genetic RNAi library screens to identify novel kDNA replication proteins [35] or cell cycle control proteins in general provide promise for the identification of novel cell cycle regulators. Recently, a systematic RNAi analysis of chromosome 1 genes identified 14 genes (∼7% genes analysed) with putative functions in nucleus and kinetoplast replication and cytokinesis [55], suggesting, by extrapolation to the whole genome, that several hundred genes may be involved in these processes. Clearly, forward genetic and proteomic approaches will be crucial in the future to identify additional components of regulatory pathways, complexes and checkpoints, particularly divergent or trypanosome-specific regulators.

## Figures and Tables

**Table 1:** Features of *T. brucei* CRKs

Protein	Accession number^a^	T_14_-Y_15_-T_161_	PSTAIRE box sequence^b^ (no. of substitutions)	N-terminal extension?	C-terminal extension?	Insertions in kinase domain?
CRK1	Tb10.70.7040	S-Y-T	EGVPCTAIREISILKE (2/16)	No	No	One 11 aa insert
CRK2	Tb927.7.7360	S-Y-T	GVPSTAVREVSLLREL (4/16)	42 aa	6 aa	No
CRK3	Tb10.70.2210	T-Y-T	EGIPQTALREVSILQE (6/16)	19 aa	No	No
CRK4	Tb08.5H5.130	T-Y-S	DGAPSTAIREIALLKV (4/16)	No	19 aa	Two large inserts (70 and 72 aa)
CRK6	Tb11.47.0031	T-Y-T	EGVPATTLREVTLLHE (6/16)	18 aa	No	Two small inserts (10 and 7 aa)
CRK7	Tb07.43M14.340	R-F-T	EGIPHMVARELLVSMR (11/16)	No	No	No
CRK8	Tb11.02.5010	S-F-T	RSLSQPTLREVILLSQ (12/16)	62 aa	19 aa	No
CRK9	Tb927.2.4510	V-Y-T	VGFPPYLLREFDLLLR (9/16)	265 aa	93 aa	Several small plus large 81 aa insert
CRK10	Tb927.3.4670	M-Y-Q	EGLPASALREVMVLKE (5/16)	18 aa	19 aa	Several, largest = 23 aa
CRK11	Tb927.6.3110	A-Q-A	RGVSEGALREATLLTL (9/16)	30 aa	49 aa	Several, largest = 30 aa
CRK12	Tb11.01.4130	T-Y-T	EGFPITSLREVIALQH (9/16)	325 aa	43 aa	Several, largest = 42 aa

The amino acid sequences of the *T. brucei* CRKs are compared to human CDK1.

a Accession numbers are given for GeneDB (www.genedb.org).

b The PSTAIRE box sequence in human CDK1 is EGVPSTAIREISLLKE.

**Table 2:** Classification of *T. brucei* cyclins

Cyclin	Alternative name	Accession no.^a^	Functional class	Functional data available?
CYC2	CycE1	Tb11.01.5660	G _1_ cyclin	Yes [12,18]
CYC3	CycB1	Tb927.6.1460	Mitotic	Yes [12]
CYC4	CycE3	Tb927.7.7170	CYC2-like	No
CYC5	CycE4	Tb10.26.0510	CYC2-like	Yes [12]
CYC6	CycB2	Tb11.01.8460	Mitotic	Yes [7,12]
CYC7	CycE2	Tb927.6.5020	CYC2-like	No
CYC8	CycB3	Tb927.7.1590	Mitotic	No
CYC9		Tb11.01.5600	Cyclin C-like	No
CYC10		Tb927.8.6340	CYC2-like	No
CYC11		Tb927.8.6350	CYC2-like	No

a Accession numbers are given for GeneDB (www.genedb.org).
